# Fear on the networks: analyzing the 2014 Ebola outbreak

**DOI:** 10.26633/RPSP.2017.134

**Published:** 2017-12-05

**Authors:** Marcelo D´Agostino, Felipe Mejía, Ian Brooks, Myrna Marti, David Novillo-Ortiz, Gerardo de Cosio

**Affiliations:** 1 Health Information and Analysis Unit Department of Communicable Diseases and Health Analysis, Pan American Health Organization Regional Office of the World Health Organization Washington, DC. United States of America Health Information and Analysis Unit, Department of Communicable Diseases and Health Analysis, Pan American Health Organization, Regional Office of the World Health Organization, Washington, DC, United States of America. Send correspondence to Marcelo D’Agostino; 2 International Consultant International Consultant Bogotá Colombia International Consultant, Bogotá, Colombia.; 3 School of Information Sciences University of Illinois, Urbana-Champagne Illinois United States School of Information Sciences, University of Illinois, Urbana-Champagne, Illinois, United States; 4 Department of Knowledge Management Bioethics,and Research, Pan American Health Organization Washington, DC. United States Department of Knowledge Management, Bioethics, and Research, Pan American Health Organization,Washington, DC, United States

**Keywords:** Hemorrhagic fever, Ebola, disease outbreaks, social media, social communication in emergencies, health communication, Internet, Enfermedad por el virus de Ebola, brotes de enfermedades, medios de comunicación sociales, comunicación social de emergencia, comunicación en salud, Internet, Doença pelo vírus Ebola, surtos de doenças, mídias sociais, comunicação social de emergência, comunicação em saúde, Internet

## Abstract

*During the 2014 Ebola outbreak, information spread via multiple platforms, including social networks and Internet search engines. This report analyzes Twitter tweets, Facebook posts, and Google trends, as well as several other Internet resources, from March – November 2014. Understanding the types of discussions, social behaviors, feelings expressed, and information shared during the Ebola outbreak can help health organizations improve communication interventions and avert misinformation and panic during health emergencies*.

*In all, 6 422 170 tweets, 83 Facebook posts, and Google search trends were integrated with 63 chronological Ebola-related events. Events that prompted a surge in tweets using #ebola were related to new cases of infection or the entry of the disease into a new goegraphic area. Most tweets were re-tweets of information provided by news agencies and official health organizations. Events related to new infections and deaths seemed to correlate with an increase of words that express fear. Google results concurred with Twitter and Facebook*.

*Data from social media activity can be used to form hypotheses about how the public responds to and behaves during public health events, prompting health organizations to adopt new strategies for communications interventions. Furthermore, a spike in activity around a topic can be used as a surveillance technique to signal to health authorities that an outbreak may be underway. It is also recommended that news agencies, which engage with the public most often, consider content review by health experts as part of their health communications process*.

## RESUMEN

Durante el brote de ébola del 2014, se difundió información por medio de varias plataformas, entre ellas las redes sociales y los motores de búsqueda de Internet. En este informe se analizan los tuits en Twitter, los mensajes publicados en Facebook y las tendencias de búsqueda en Google, así como varios recursos más en Internet, en el período comprendido entre marzo y noviembre del 2014. La comprensión de los tipos de conversaciones, el comportamiento social, los sentimientos expresados y la información transmitida durante el brote de ébola puede ayudar a las organizaciones de salud a mejorar sus intervenciones en materia de comunicación y evitar la información incorrecta y el pánico que se pueden propagar durante las emergencias de salud.

En total, se integraron 6 422 170 tuits, 83 mensajes de Facebook y las tendencias de búsqueda en Google con 63 eventos cronológicos relacionados con ébola. Los eventos que dieron lugar a un incremento de los tuits con la etiqueta #ebola estaban relacionados con nuevos casos de infección o la entrada de la enfermedad en una nueva zona geográfica. La mayor parte de los tuits eran reenvíos de información suministrada por las agencias de noticias y las organizaciones de salud oficiales. Los eventos relacionados con nuevas infecciones y defunciones parecían guardar correlación con un aumento del uso de palabras que expresaban temor. Los resultados de Google coincidían con Twitter y Facebook.

Se pueden emplear datos provenientes de la actividad de las redes sociales para formar hipótesis sobre el modo en que el público responde a los eventos de salud pública y en que se comporta durante ellos, e incitar a las organizaciones de salud a que adopten nuevas estrategias para las intervenciones en materia de comunicación. Además, se pueden usar los aumentos de la actividad en torno a un tema como técnica de vigilancia para señalar a las autoridades de salud que es posible que haya un brote. Se recomienda también que las agencias de noticias, que interactúan con el público con más frecuencia, consideren la posibilidad de contar con expertos en salud para examinar los contenidos en el marco de su proceso de comunicación para la salud.

## RESUMO

No transcurso do surto de Ebola de 2014, a informação se espalhou por diversas plataformas como as redes sociais e os mecanismos de busca da internet. Este artigo examina as mensagens no Twitter, postagens no Facebook e tendências no Google e outras mídias digitais no período de março a novembro de 2014. Entender as formas de discussões, comportamentos sociais, sentimentos expressos e informações partilhadas no decorrer do surto de Ebola pode contribuir para melhorar as intervenções em comunicação por parte das organizações de saúde e evitar a desinformação e o pânico em situações de emergência em saúde.

Ao todo, 6.422.170 mensagens no Twitter, 83 postagens no Facebook e tendências de busca no Google foram correlacionadas a 63 eventos cronológicos relativos ao surto de Ebola. Os eventos que suscitaram um aumento no número de mensagens no Twitter com o hashtag #ebola foram a ocorrência de novos casos de infecção ou o surgimento da doença em outras áreas geográficas. Grande parte da atividade no Twitter consistiu no reenvio de mensagens com informações fornecidas pelas agências de notícias e organizações oficiais de saúde. Os eventos relacionados a novas infecções e mortes aparentemente se correlacionaram ao uso de um maior número de palavras que expressam temor. Os resultados do Google coincidiram com os do Twitter e Facebook. A análise da atividade nas mídias sociais permite formular hipóteses sobre a reação e o comportamento do público em situações de risco em saúde pública, motivando o uso de novas estratégias de comunicação por parte das organizações de saúde. Um pico de atividade em torno de um assunto pode ser um recurso de vigilância usado pelas autoridades sanitárias para sinalizar a possível ocorrência de um surto. Também se recomenda às agências de notícias, por estarem em contato constante com o público, integrar a análise do conteúdo por especialistas em saúde ao processo de comunicação de assuntos em saúde.

Although the evidence is fragmented and some of its real effects are contradictory, it is clear that fear, trust, love, and skepticism influence the behavior of individuals participating in social networks (1, 2). Given this dynamic, social networks provide unlimited opportunities to improve public health interventions (3). However, to harness the power of social networking, an effective online communications strategy requires an unambiguous understanding of the Information Society (4).

The level of penetration, use, and popularity of information and communication technologies among the world’s population creates agile bridges of information on a diverse range of events that are of global concern or of particular interest. Disasters, movies, economic debacles, sporting events, political meetings, and even music videos come from and go to all possible corners. Once there is an event of interest, networks are flooded with comments, memes (a viral digital content that acts as cultural and social symbol and idea), photos, blogs, and videos. These posts can be “liked” and shared millions of times, sometimes changing their use, and therefore, their meaning.

**FIGURE 1.**
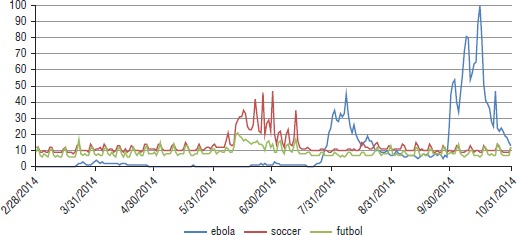
Worldwide Google search trends on “Ebola” in English and Spanish, compared to “soccer” and “futbol” 28 February – 31 October 2014

All this engagement does not imply that the content to which people react is of good quality. Indeed, separating whatis true from what is false is an arduous task, one that the general public may not always be able to perform accurately. Within public health, there are topics of interest whose social media activity is generally stable over time. There is another set of topics that has a more defined time effect; some arise and stabilize quickly, such as those associated with natural disasters; while others develop slowly, and seem to grow exponentially, such as those associated with major epidemics. Polgreen and colleagues have asserted that the Internet has “dramatically changed how people search for medical information…especially about infectious diseases…Thus, the frequency of Internet searches may provide information regarding infectious disease activity (5).”

Although there are and always will be, situations that cause fear in society, this report shows that fearful reactions to fears or in individuals normally occur when the situation might affect them personally. In public health emergencies, such as the 2014 Ebola out-break, it is an enormous challenge to communicate uncertainty without igniting fear and undermining public trust in health authorities (6). Figure 1 compares the term “Ebola” (a term associated with fear) to “soccer” and “futbol”(associated with pleasure), and shows that only in two situations did fear out-number pleasure. On 2 August 2014, a United States missionary physician infected with Ebola in Liberia was flown to Atlanta, Georgia (United States) for treatment. On 8 October 2014, Duncan, the first person diagnosed with Ebola in the United States, died in a hospital in Dallas, Texas. The Government of the United States ordered that passengers arriving from West Africa at five major airports be screened for fever.

This study aims to support governments, mass media, and health-related institutions in developing strategies that explicitly seek to generate effective communication campaigns with a special focus on online tools, such as social networks and virtual libraries. It also seeks to reduce the know–do gap on the use of information and communication technologies for the prediction of social behavior (7, 8).

## EBOLA AND SOCIAL MEDIA

### Chronology of key events

A chronology of 63 key events from March – November 2014 associated with the outbreak of Ebola was developed based on data published by Reuters (9). Among these key events were the first reported cases, the infected health workers returning to their home countries, first treatments and potential vaccines, news from known leaders, and so on.

The sources of information chosen to gather data for this study were: Google (Mountain View, California, United States); Wikipedia (San Francisco, California, United States); Facebook (Menlo Park, California, United States) and Twitter (San Francisco, California, United States); and such news, blogs, and analytical tools as Symplur (Riviera Beach, Florida, United States), Topsy (San Francisco, California, United States), and Hashtags.org (Chicago, Illinois, United States). Public tweets and re-tweets (RTs) and Facebook posts that included the hashtag “#ebola” were collected; their distribution was determined for the study period and integrated with the chronology. The most mentioned Twitter profiles were determined, those using #ebola the most, and those with the highest number of impressions. Major peaks were determined, and transcriptions of the tweets around these peaks were analyzed to find the most common topics, as well as the most common words. RTs and content with non-related words (identified as noise) were not considered.

### Data management

A total of 6 422 170 tweets and 142 Facebook posts were retrieved and organized in several Microsoft Excel™ (Microsoft Corp., Redmond, Washington, United States) spreadsheets. The chronological lists of events was integrated with the chronology of tweets and Facebook posts.

#### Twitter.

**FIGURE 2.**
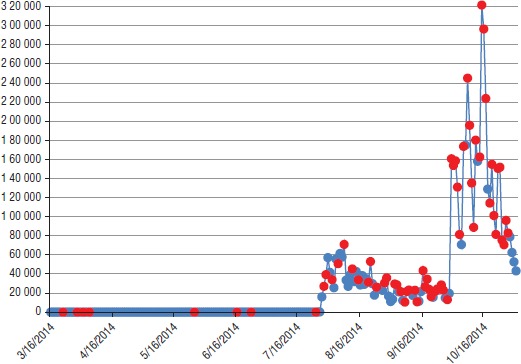
Worldwide tweets using #ebola integrated with 63 Ebola-associated chronological events (in red dots) in the time range, 16 March – 16 October 2014

All tweets were grouped into weekly time ranges, from Sunday to Sunday. Through the same platform, the following information was determined: total number of impressions, total number of tweets, total number of participants, average tweets per hour, and average tweets per participant. The number of tweets and their timeline were charted in order to identify areas for further analysis, as follows: (a) first area, 424 tweets from 16 March – 24 July 2014; (b) second area, 1 863 764 tweets from 24 July – 29 September 2014; and (c) third area, 4 555 982 tweets from 29 September – 2 November 2014. The accounts with the highest influence during those periods were determined by the number of mentions. The chronology is integrated with the timeline of tweets in one chart and for each area. Every number represents one specific event in the chrochronology (Figure 2).

#### Facebook.

Using the Facebook search function, posts that included the hashtag #ebola were identified. The search capabilities did not allow a time range to be specified; so, the results of the search conducted on 10 November 2014 at 12:57 p.m. were scrolled through to collect all posts from 16 September – 10 November 2014.

#### Search trends.

**FIGURE 3.**
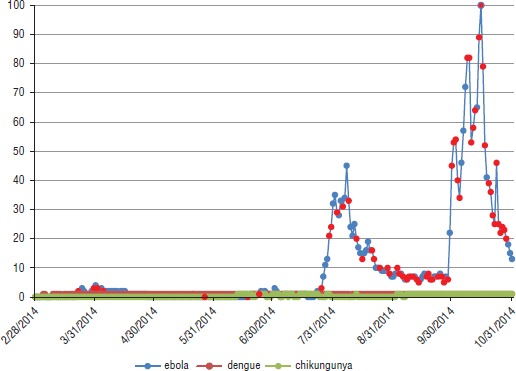
Worldwide Google trends for Ebola, dengue, and chikungunya with 63 Ebola-associated chronological events (in red dots) in the time range, 28 February – 31 October 2014

Google Trends was used to determine trends in relation to other topics, such as dengue and chikungunya (Figure 3). Similarly, the number of results in other sources, such as SlideShare (LinkedIn Corp., Mountain View, California, United States), and LinkedIn were compiled. The Ebola trend was also referenced with the chronology.

## RESULTS

### Twitter

By preliminary analysis, the events that prompted an increase in the number of tweets using #ebola were those related to new cases of infection or the entry of the disease into a new geographic area. Most tweets were RTs of information provided by news agencies and official health organizations. News agencies significantly exceeded health organizations in terms of RTs, and thereby, influence. The most common language was English, as expected since most Twitter users predominantly reside in Englishspeaking areas. The only time a language (Spanish) surpassed English in Twitter usage was when the first case of Ebola occurred in Europe (in Madrid, Spain).

The number of tweets from official health organizations containing facts and recommendations decreased over time, but there was an increase in information about official reports on the number of cases and deaths. Finally, it was evident that events related to new infections and deaths seem to correlate with an increase in the use of words that express fear and worry. The use of words that users associated with symptoms of Ebola increased with time, but do not seem related to specific events (Table 1).

### Facebook

The trend on Facebook behaved somewhat differently from the trend on Twitter. Moderate peaks were found in October, with a surge of high peaks in November; however, no events were registered for November, at least not according to the constructed chronology. This incongruence must have been related to Facebook’s search functionality, which displays a greater number of posts closer to the time when the search is performed. By eliminating the November posts from the timeline, the Facebook and Twitter trends and associated events were more closely aligned.

**TABLE 1.** Content analysis of tweets in comparison with ebola-related topics based on the chronology of events.

Area	Characteristics of the news	Language use	Most common topics
I	Suspicion of symptoms	Baseline	- Consequences of the disease in the body. -Spread of the disease to other countries.
I	Launch of worrying reports	- Languages: Arabic, French, German, Italian, and Korean - Use of phrase “out of control”	- Ability of other African states to control the entrance of Ebola. - Mortality rate of Ebola. - Relation of the Ebola outbreak with other, seemingly unrelated events.
II	First case in Nigeria	Use of “#ebola is real,” “protect yourself” Use of words such as “worse,” “worst,” “worry,” and “myths”	- Likelihood of Ebola hitting their countries. - The need to include other health aspects within the Ebola trend of information. - Touching others who might be infected. - Call for keeping panic under control.
II	First entry into United States of America	- Use of words like “worry,” “worse,” “fear,” “risk,” and “scare,” and symbols such as “:(“ - Use of the word “God” - Use of the word “vaccine”	- Impact of the presence of Ebola in Africa on African stereotypes. - Demand for action. - Using the trend of Ebola to hide other important global topics. - Ebola hitting the Middle East. - Ebola hitting other continents.
II	International aid initiatives	Use of the words “fear,” “worse,” “worry,” “frightening,” “panic”	- Excess of information related to Ebola. - Ebola mutations. - Airborne contagion. - Locals are “left to die;” are “flown out.” - Doubt of the capabilities of the World Health Organization
II	First case in the United States	Use of words “fear,” “worse,” “worry,” “frightening,” “panic”	- Ebola-infected person in United States. - Increase in Ebola facts especially from the White House.
III	First case in Spain First case in France	- Use of words “fear,” “worse,” “worry,” “frightening,” “panic” - Tweets in Spanish - Tweets in Frenchç	- Symptoms that users associate with Ebola. - Other symptoms. - Airborne disease. - Conspiracy attitudes. - Use of facemasks.
III	First case in United States–and spread declaration	Use of words “fear,” “worse,” “worry,” “frightening,” “panic”	- Symptom that users associate with Ebola. - Airport screenings to detect symptoms. - Contagion by health workers. - Living in areas with cases. - Little focus on African patients. - Virus considered manufactured.

### Google Trends

The results of Google Trends, expressed in relative measures from 0 – 100, were similar to those of Twitter and Facebook. The same events correlate to similar peaks. In comparison with other infectious diseases, Ebola notably surpassed chikungunya and dengue.

### Others

Table 2 summarizes the Ebola-resulted results for various other online services, such as YouTube (Alphabet Inc., San Bruno, California, United States), SlideShare, LinkedIn, other Google search applications, including Google web search, Google news search, Google search videos, Google search books, Twitter, and Hashtags.org.

## DISCUSSION

Beyond simply accessing information, the population is now active in the creation of content: not only does news travel almost instantaneously from one side of the world to the other, but thanks to social networks, reactions also come in from its recipients.

The way media communicates and the way each person reacts to an event is different and conditioned by unique factors and the local circumstances of the place in which the event occurred. This complex interaction creates a challenge for health organizations that are striving to effectively and efficiently communicate with an affected population, whether it is to mobilize, prevent, reduce fear, or encourage. The need to listen in detail to the population before deciding how to communicate, what to communicate, through what media, and to what audience is imperative.

The 2014 West Africa Ebola epidemic was an event that initially developed at a slow pace, without great impact among people—only among health agencies. Eventually, it became a topic of massive interest; Ebola news was distributed through multiple channels and sources, simultaneously causing a social media response by the population, some seeking information to help them make certain decisions, some to make statements, and some to express dissent or concern (10). Some authors have asserted that,

… at the early stages of an outbreak, informal sources can be indicative, not just that an outbreak is occurring, but can highlight disease dynamics through estimation of a key epidemic parameter, the reproductive number. Social and news media, such as from HealthMap and Twitter are a cost-effective data source (11).

Data from social media can be used to draw hypotheses about how people and institutions behave in relation to public health events. In the case of epidemics, panic-related behavior in affected communities might be closely related to the type and frequency of words used; therefore, communication by health organizations should adapt dynamically and rapidly to the type of panic and the increased volume of data. Data can be used as a proxy for a major panic outbreak. An in-depth analysis of the impact of their social media efforts on the population should be an integral part of every health organization’s communication strategies.

**TABLE 2.** Ebola by the numbers: worldwide content available on 16 March – 2 November 2014

Tool	Description	Ebola content	Additional information
YouTube	YouTube allows billions of people to discover, watch, and share originally created videos.	806 000 videos	Search term: Ebola outbreak 2014
SlideShare	SlideShare has grown to become the world’s largest community for sharing presentations and other professional content.	5 345 presentations	- 3 352 in English - 8 79 ain Spanish -5 44 in Portuguese -161 in French
LinkedIn	LinkedIn is a business-oriented social networking service, principally used for professional and career purposes.	4 338 posts	- 70 professional groups discussing Ebola. - 70 people self-identified as Centers for Disease Control and Prevention (United States).
Google web search	Google is one of the five most popular search engines in the world.	72 500 000 results	Search term: Ebola outbreak 2014
Google news search	Google News is a computer-generated news site that aggregates headlines from news sources worldwide.	8 910 000 results	Search term: Ebola outbreak 2014
Google search videos	Google Videos is a video search engine from Google.	25 200 000 results	Search term: Ebola outbreak 2014
Google search books	Google Books is a service that searches the full text of books and magazines that Google has scanned.	1 250 results	Search term: Ebola outbreak 2014
Twitter	Twitter is an online social networking service that enables users to send and read short 140-character messages called “tweets.”	7 605 318 tweets	Tweets on Ebola. Last 30 days.
#ebola hashtag	On social media sites such as Twitter, a word or phrase preceded by a hash or pound sign (#) and used to identify messages on a specific topic.	1 281 731 tweets using #ebola hashtag	#ebola. Last 30 days.

This analysis also showed that in many cases, news agencies have more engagement with the public than do health organizations; given this, some level of content surveillance by health organizations is needed. At the same time, social media data—unstructured data—requires sizable computational capacity to conduct proper analysis and to establish sound conclusions. This study faced a considerable methodological challenge, which was confronted by selecting small samples, but which led to difficulties with generalizing the conclusions.

Health institutions must continue developing communication and communication-based surveillance strategies that explicitly seek to generate more impact as influencers of information networks— especially in relation to news agencies— or, to at least insure that news agencies are using health institutions as important sources. Strategies must be applied to both ongoing and sudden public health issues, using shared information as a rich source for evaluating behavior expressed on social media. Communication and community outreach should be seen as vital components of an integrated public health response plan that is based on established science. When health communications initiatives are developed independently from other parts of the response plan, they may become marginalized and ineffective (12).

Notwithstanding, the unstructured nature of these data proves a challenge for fast and accurate analysis. For qualitative assessments, in particular, data needs to be in a readable and decipherable format. However, even in raw form, social media data can provide key insights into people’s attitudes, which can in turn allow for fine-tuning of communication strategies and improved support for socio-epidemiological assessments.

### Limitations

This study considered only tweets and Facebook posts that included the hashtag “#ebola,” which may have reduced the total possible number of tweets and posts of interest. Since the quantitative approach includes all tweets and re-tweets, it is likely that some repeat or the use of unrelated words may also have biased the conclusions. A strategy to avoid noise in content should be included in further analysis, particularly for the quantitative analysis. This noise avoidance is carried out only in the qualitative analysis. On the other hand, it is not possible to obtain all Facebook posts of interest through its search engine; and the results may have been filtered and/or customized by Facebook, which limited the real study scope for this social network.

## CONCLUSIONS

Clearly, communication campaigns in the current context of the “Information Society” should have a special focus on online tools, social networks, and strategic content development. Health communication strategies should include creating and providing high quality content and also verifying content on websites such as Wikipedia, monitoring discussions and questions on LinkedIn, sharing all presentations related to diseases and health issues, such as Ebola on SlideShare, and improving web resources, including open libraries, mailing lists, among others.

Further research is needed to determine if unstructured data taken from social networks can be trusted to predict epidemics and how these nontraditional sources and tools can best be used for monitoring future epidemics and reducing fear among the population.

Facts and recommendations on how to manage disease, prevent new infections, and identify symptoms should be continuously delivered in order to raise awareness and keep users, other health organizations, and news agencies informed. The content of tweets should be written in a way that aims to control fear and paranoia among people. Tweets with valid and complete information and recommendations, rather than links to further information, are preferred. The most influential news and health information sources should be continuously reviewed for accuracy. Finally, health organizations must be in front of news agencies, developing specific and continuous Internet communication and strategies for knowledge dissemination in order to become the most influential source of public health information.

## Disclaimer.

Authors hold sole responsibility for the views expressed in the manuscript, which may not necessarily reflect the opinion or policy of the *RPSP/PAJPH* and/or PAHO.
